# Genome sequence of ground tit *Pseudopodoces humilis *and its adaptation to high altitude

**DOI:** 10.1186/gb-2013-14-3-r29

**Published:** 2013-03-28

**Authors:** Qingle Cai, Xiaoju Qian, Yongshan Lang, Yadan Luo, Jiaohui Xu, Shengkai Pan, Yuanyuan Hui, Caiyun Gou, Yue Cai, Meirong Hao, Jinyang Zhao, Songbo Wang, Zhaobao Wang, Xinming Zhang, Rongjun He, Jinchao Liu, Longhai Luo, Yingrui Li, Jun Wang

**Affiliations:** 1 BGI-Shenzhen, Beishan Industrial Zone, Yantian District, Shenzhen 518083, China; 2 Department of Biology, University of Copenhagen, DK-1165 Copenhagen, Denmark; 3 King Abdulaziz University, Abdulla Alsulaiman Road, Jeddah 21589, Saudi Arabia

**Keywords:** genome, high-altitude adaptation, phylogeny

## Abstract

**Background:**

The mechanism of high-altitude adaptation has been studied in certain mammals. However, in avian species like the ground tit *Pseudopodoces humilis*, the adaptation mechanism remains unclear. The phylogeny of the ground tit is also controversial.

**Results:**

Using next generation sequencing technology, we generated and assembled a draft genome sequence of the ground tit. The assembly contained 1.04 Gb of sequence that covered 95.4% of the whole genome and had higher N50 values, at the level of both scaffolds and contigs, than other sequenced avian genomes. About 1.7 million SNPs were detected, 16,998 protein-coding genes were predicted and 7% of the genome was identified as repeat sequences. Comparisons between the ground tit genome and other avian genomes revealed a conserved genome structure and confirmed the phylogeny of ground tit as not belonging to the Corvidae family. Gene family expansion and positively selected gene analysis revealed genes that were related to cardiac function. Our findings contribute to our understanding of the adaptation of this species to extreme environmental living conditions.

**Conclusions:**

Our data and analysis contribute to the study of avian evolutionary history and provide new insights into the adaptation mechanisms to extreme conditions in animals.

## Background

The Qinghai-Tibet Plateau (QTP), known as 'the roof of the world', has become the focus of many biological studies. Many aboriginal animals, such as the Tibetan antelope, sheep and yak, and even humans (Tibetans), survive there under extreme environmental condition, including reduced oxygen supply and freezing temperatures. Similar scenarios can be found in the Andean Altiplano (guinea pig) and Ethiopia's Simien Plateau (Simien fox), the two other highland plateaus on Earth. Scientists have begun to explore the mechanisms that underlie organisms' adaptation to high altitudes. Some genes related to high-altitude adaptation in Tibetans [1-3] and in yaks [4] have been detected. Some studies have investigated birds at high altitudes and across altitude gradients. Bulgarella *et al*. [5] reported the combined effects of selection and population history on levels of population divergence for the crested duck (*Lophonetta specularioides*), which is distributed across an altitudinal gradient and in which selection for hypoxia resistance may have played an important role. Multilocus coalescent analysis revealed hemoglobin differentiation between low- and high-altitude populations of crested ducks [5]. In the bar-headed goose, adaptations in mitochondrial enzyme kinetics and oxygen transport capacity through the molecular evolution of cytochrome C oxidase were thought to contribute to the exceptional ability of these geese to fly at high altitudes [6]. However, in the QTP native avian species *Pseudopodoces humilis *(common name, the ground tit), the genetic mechanisms of high-altitude adaptation have never been studied. These birds exhibit morphological, physiological, and behavioral adaptations to life in this open habitat, including pale cryptic plumage; a long decurved bill for probing in crevices among rocks or in the ground; long legs for terrestrial locomotion; and nesting in burrows of small mammals, rock crevices, or building holes [7]. Ground tits may have adapted to high-altitude conditions via different genes or functional pathways.

There has also been controversy about the phylogeny of the ground tit. It was first classified as belonging to the *Pseudopodoces *genus [8], but a recent phylogenetic analysis indicated that it was not a member of the Corvidae family [9]. Recently, the ground tit was determined to belong to the Paridae family, according to an analysis of its mitochondrial genome and some of its nuclear genes [7]. However, because the whole genome sequence of the ground tit and other close avian species are still unavailable, no definite conclusion has yet been reached.

To date, very little genetic or other information about the ground tit has been published. To explore the genetic mechanisms of the ground tit's adaptation to high altitude, we sequenced its genome and identified 16,998 protein coding genes. The results provided an insight into the evolutionary relationships among avian species, and also between and within Passeriformes (including *P. humilis *(ground tit) and *T. guttata *(zebra finch)) and between Galliformes (including *G. gallus *(chicken) and *M. gallopavo *(turkey)). In addition, we aimed to identify some of the factors that contribute to the high-altitude adaptation of the ground tit, which may help in understanding the mechanisms used by other species in the QTP highlands.

## Results

### Sequencing and assembly

**Figure 1:**
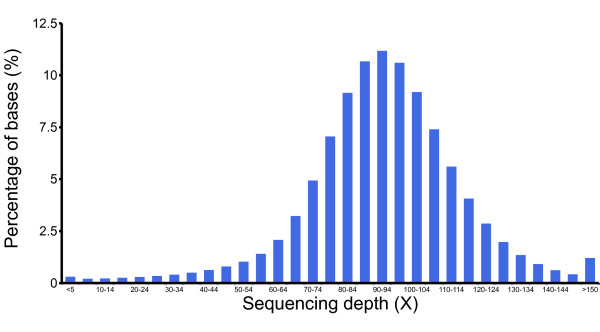
**Sequencing depth of the ground tit genome assembly**. The sequencing depth was measured by initially mapping all the raw reads to the assembly, and then calculating the number of reads for each base. More than 99% of the assembly sequences were covered 20× at least, indicating high accuracy at the nucleotide level.

The ground tit genome was sequenced on the Illumina HiSeq platform. We obtained 184.5 Gb of raw sequence from several size-ranked libraries (Additional file 1, Table S1). Then, a series of filtering steps was undertaken to filter the low-quality sequencing reads. After assembling the retained 119.0 Gb clean data using SOAPDenovo [10], 1.04 Gb of assembled genome sequence, similar in size of other bird genomes, was generated. The N50 lengths of the scaffolds and contigs were 16.3 Mb and 164.7 Kb, respectively (Additional file 1, Table S2), indicating that this assembly may contain a significantly high number of complete protein-coding genes (Table 1). The average sequencing depth of the ground tit assembly was 96×, and 99% of the assembly had a coverage of at least 20× (Figure 1), ensuring high accuracy at the nucleotide level. Based on the k-mer coverage value [11], the genome size was estimated to be 1.1 Gb, indicating that 95.4% of the genome was covered in our assembly.

**Table 1:** Details of the assembly parameters for selected avian species

Species	Assembly size (Gb)	Estimated genome size (Gb)	N50 Scaffold length (Mb)	N50 contig length (Kb)
Chicken	1.05	1.06	7	36
Zebra finch	1.2	\	10	39
Turkey	1.04	1.05	1.5	12.6
Ground tit	1.04	1.1	16.3	164.7

### Genome annotation

We analyzed the ground tit genome for repeats and GC content, and found that the overall GC content was 41.7%, which is similar to that in other birds and higher than in humans (Additional file 1, Figure S1). Based on searches of Repbase (version 2011-09-20) and de novo repeat libraries (Additional file 1, Table S3), we found that 7% (72.6 Mb) of the genome comprised repeat sequences, which is slightly less than in the chicken and zebra finch genomes (Additional file 1, Table S4).

SOAPAlign [12] was used to align the reads onto the genome, and SOAPsnp [13] was then used in a variation analysis to identify heterozygous alleles. A total of 1,723,688 single nucleotide polymorphisms (SNPs) were identified across the whole genome, representing a heterozygous ratio (the average nucleotide diversity between the chromosome pairs of diploid genome) of 1.68 × 10^-3^. We found that 20,312 of the SNPs were located in potential coding regions, giving an average heterozygous allele ratio of 0.85 × 10^-3^, almost half that of the whole genome, indicating that coding regions were highly conserved.

We then annotated the candidate protein-coding genes by manually integrating *de-novo *gene predictions, expressed sequence tag (EST) evidence from closely-related species, and homolog information (Additional file 1, Figures S2 and S3, and Table S5; Methods). These analyses identified 16,998 potential coding genes, 98.9% of which were annotated with a function based on BLAST hits to various databases (Additional file 1, Table S6; Methods). The conservation of gene sequences between the ground tit and other birds and mammals correlated well with the conservation at the amino acid sequence level. The avian amino acid sequences shared higher amino acid sequence identity with mammalian sequences than with chicken sequences (Additional file 1, Figure S4). We also annotated 193 microRNAs, 116 tRNAs, eight rRNAs and 239 small nuclear RNAs (snRNAs) (Additional file 1, Table S7).

### Genomic analyses and comparison across the bird phylogeny

#### Syntenic map between the ground tit, zebra finch, and chicken

**Figure 2:**
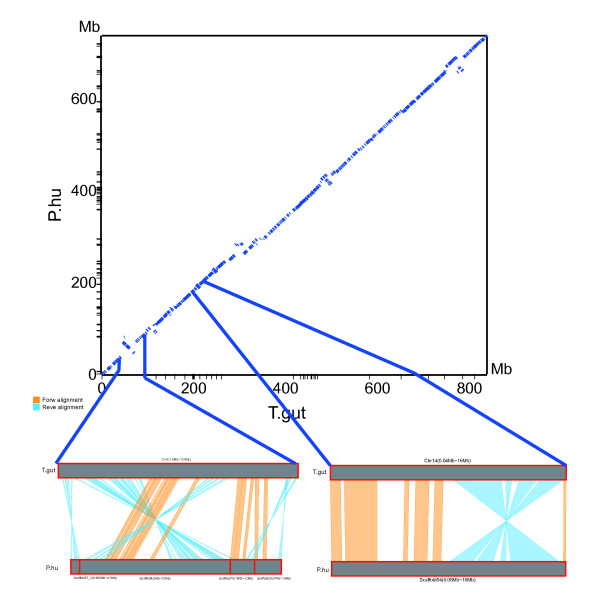
**Micro-synteny between the zebra finch and ground tit genomes**. The X-axis represents the zebra finch (T. gut) chromosomes and the Y-axis represents the ground tit (P. hum) scaffolds. The 'dots' represent ortholog gene pairs between the two genomes. The scaled-up regions of the dot plot show two obvious chromosome inversions. In the right most up-scaled figure, one part of the scaffold54 sequence of P. hum is aligned in the forward orientation (colored in orange) to that of Chr4 of T. gut, while the other part of the scaffold54 sequence were aligned in the reverse orientation (colored in blue sky) to that of Chr4 of T. gut, indicating that there was a chromosome inversions in P. hum compared with T. gut. A similar inversion is shown on the left.

To identify chromosomal evolutionary trends between the zebra finch and ground tit, we mapped the ground tit scaffolds onto the zebra finch chromosomes. No genetic map for the ground tit was available; therefore, the scaffolds could not be assigned to different linkage groups. Accordingly, we anchored the scaffolds (with lengths >10 kb) to the zebra finch chromosomes according to the orthologous gene pairs identified by MCscan [14]. We found a highly conserved syntenic relationship between the ground tit scaffolds and the zebra finch chromosomes, except for some obvious chromosome inversions in the ground tit (Figure 2). This result highlighted the high degree of conservation between the two bird genomes. We then mapped the ground tit scaffolds against chicken chromosomes, and obtained a similar result (Additional file 1, Figure S5). In general, the high level of synteny between the ground tit scaffolds and the zebra finch and chicken chromosomes suggested that avian genome structure is largely conserved.

#### Three-way species alignment and ground tit phylogeny

To further investigate the evolutionary relationships between chicken, zebra finch, and ground tit, the three genomes were aligned using Lastz/Multiz [15,16]. We found that 37% of the chicken genome, 74.7% of the zebra finch genome, and 88.6% of the ground tit genome could be aligned and 400 Mb was shared by all three genomes (Additional file 1, Figure S6). This finding correlates with the expected phylogenetic relationship of these three species.

**Figure 3:**
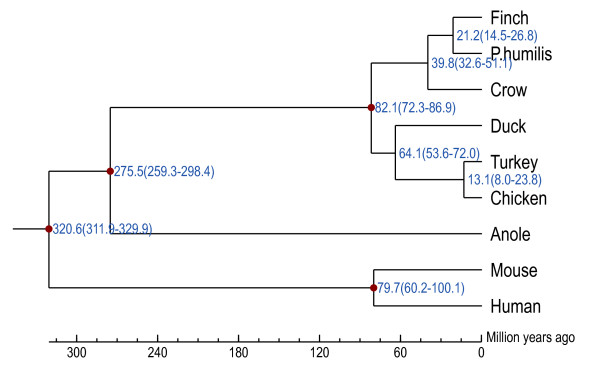
**Phylogenetic tree reconstructed using all single-copy orthologs from nine species**. The scale at the bottom of the figure represents the divergence time. The red dots represent the divergence time and its range (in brackets) between two branches.

We determined the phylogeny of ground tit using gene orthologs from nine species (zebra finch, ground tit, crow, duck, turkey, chicken, anole, human, and mouse). The ground tit was more closely related to the zebra finch (family Estrildidae)(Figure 3, Methods).

#### Lineage-specific expansion/contraction of protein-coding gene families

Frequent turnover of gene copy number has been proposed as a major mechanism underlying the adaptive divergence of closely-related species [17-20]. To assess gene copy numbers in the ground tit genome, we looked at the expansion and contraction of protein-coding gene families, which were then assigned different Gene Ontology (GO) terms [21]. The expansion of the olfactory receptor family (GO:0004984, olfactory receptor activity; *P *< 0.01, Fisher exact test) has been reported to be a shared characteristic of the Aves class [22], and the expansion of the keratin family, which comprises the major structural proteins of avian feathers, claws, and scales, has been noted in ground tit and chicken [23]. We identified 203 gene families that were substantially expanded in the ground tit compared with other birds (Table 2; Additional file 1, Figure S7). For example, digestive enzymes (GO:0004252, serine-type endopeptidase activity, *P *< 0.01; GO:0006508, proteolysis, *P *< 0.01) and glucose metabolism enzymes (GO: 0004396, hexokinase activity, *P *< 0.01; GO:0006096, glycolysis, *P *< 0.05) appeared to have undergone significant gene family expansions in the ground tit. We also identified a KEGG pathway [24] involved in dilated cardiomyopathy (map05414) that was enriched for significant gene family expansions (*P *< 0.01; Additional file 1, Table S8). These species-specific gene copy number variations indicated that the extreme cold and hypoxic conditions of the high altitude habitats of the ground tit might impose specific metabolic energy and/or cardiac functions on the ground tit [25,26]. We also identified eight gene families that had undergone significant contraction in ground tit compared with other avian species (Additional file 1, Tables S9 and S10).

**Table 2:** GO enrichment analysis of significantly expanded gene families in the ground tit genome

GO_ID	GO_Term	GO_Class	*P *value	Adjusted *P *value
GO:0001669	Acrosomal vesicle	CC	3.36E-35	7.00E-33
GO:0045095	Keratin filament	CC	2.33E-19	6.93E-18
GO:0004252	Serine-type endopeptidase activity	MF	6.33E-19	1.65E-17
GO:0005198	Structural molecule activity	MF	4.55E-11	8.60E-10
GO:0006508	Proteolysis	BP	7.79E-11	1.25E-09
GO:0044430	Cytoskeletal part	CC	1.93E-09	2.87E-08
GO:0005044	Scavenger receptor activity	MF	2.22E-08	2.31E-07
GO:0043229	Intracellular organelle	CC	1.13E-06	1.02E-05
GO:0003956	NAD(P)+-protein-arginine ADP-ribosyltransferase activity	MF	8.31E-06	7.20E-05
GO:0043565	Sequence-specific DNA binding	MF	9.53E-06	7.93E-05
GO:0007156	Homophilic cell adhesion	BP	2.35E-05	1.81E-04
GO:0004396	Hexokinase activity	MF	4.08E-05	2.92E-04
GO:0003700	Sequence-specific DNA binding transcription factor activity	MF	4.58E-05	3.07E-04
GO:0006471	Protein ADP-ribosylation	BP	7.07E-05	4.59E-04
GO:0004984	Olfactory receptor activity	MF	2.71E-04	1.57E-03
GO:0016459	Myosin complex	CC	6.88E-04	3.67E-03
GO:0006096	Glycolysis	BP	4.39E-03	2.03E-02
GO:0043234	Protein complex	CC	6.09E-03	2.75E-02
GO:0043231	Intracellular membrane-bounded organelle	CC	9.89E-03	3.67E-02
GO:0003774	Motor activity	MF	1.24E-02	4.21E-02
GO:0004872	Receptor activity	MF	1.46E-02	4.76E-02
GO:0005086	ARF guanyl-nucleotide exchange factor activity	MF	1.55E-02	4.85E-02
GO:0032011	ARF protein signal transduction	BP	1.55E-02	4.85E-02

Gene families were classified into GO categories or descendant category. The significance of the enrichment was calculated using a Fisher's exact test following a conservative correction for multiple testing (false discovery rate <0.05, Holm's correction).

BP, biological process; CC, component; MF, molecular function.

#### Rapid and slow evolutionary changes in the molecular function category within Passeriformes

To detect the GO molecular function categories that had undergone rapid or slow evolution, we searched for functionally related genes with exceptionally high or low selection constraints in the ground tit and zebra finch. For categories with at least 20 genes, the ratio of the number of non-synonymous substitutions per non-synonymous site (Ka) to the number of synonymous substitutions per synonymous site (Ks) (Ka/Ks) was calculated by concatenating the gene sequences. To identify extreme outliers, the category-specific ratios were compared with the average across all orthologs in the ground tit and zebra finch using a metric based on the binomial test (Methods). The numbers of observed outliers below a specific threshold (test statistic <0.001) were then compared with the expected distribution of outliers given randomly permuted annotations.

Twenty-two categories showed elevated Ka/Ks ratios at the specified threshold in the ground tit compared with the zebra finch (Additional file 1, Table S11). Based on 10,000 repeated trials after randomly permuted annotations, 14 would be expected by chance, indicating that eight of the 22 categories may have undergone significantly accelerated evolution relative to the genome-wide average (*P *< 0.0556). One category of interest was that for DNA repair (GO0006281, *P *= 6.60E-43), because genes related to DNA repair might be related to the high-dose UV environment of the ground tit (Additional file 1, Table S12). Fifty-five categories showed significantly low Ka/Ks ratios (compared with 19 expected by chance; *P *< 0.0001). These categories mainly comprised ion transport and signal transduction processes, which seem to be under stronger-than-average purifying selection in the ground tit (Additional file 1, Table S13).

#### Positively selective genes

Genome-wide scans for positively selected genes in birds have provided insights into the dynamics of genome evolution, the genetic basis of differences between species, and the functions of individual genes. To reveal potential targets of positive selection in the ground tit, we analyzed 6,625 1:1 gene orthologs from the ground tit, zebra finch, chicken, and turkey by genome alignment. Likelihood ratio tests based on a branch site model revealed 34 positively selected genes (Methods; Table 3), which were enriched for roles in signal transduction and development, including 'beta-adrenergic receptor kinase activity', 'tachykinin receptor activity', 'steroid biosynthetic activity', 'hexosamine biosynthesis activity', and 'cardiac muscle tissue development'.

**Table 3:** List of positively selected genes identified in the ground tit genome

Gene symbol	Gene description	*P *value
UAP1	UDP-N-acteylglucosamine pyrophosphorylase 1	1.30E-03
ST6GALNAC1	Alpha-N-acetylgalactosaminide alpha-2,6-sialyltransferase	1.20E-03
PIGW	GPI anchor biosynthetic process	1.00E-03
SNAP29	SNA transporter activity	9.00E-04
HSD17B12	Hydroxysteroid (17-beta) dehydrogenase 12	8.00E-04
SLC26A5	Sensory perception of sound	8.00E-04
MRPL27	Mitochondrial ribosomal protein L27	8.00E-04
ZFP64	Regulation of transcription	7.00E-04
TYMS	Thymidylate synthase	7.00E-04
ATL2	Atlastin GTPase 2	6.00E-04
VRK1	Protoprotein amino acid phosphorylation	6.00E-04
TACR1	Tachykinin receptor 1	3.00E-04
CLDN1	Claudin 1	3.00E-04
C13orf27	Hydrolase activity	3.00E-04
GDF7	Growth factor activity	2.00E-04
INPP5F	Inositolpolyphosphate-5-phosphatase F	2.00E-04
CPM	Carboxypeptidase M	9.25E-05
AQP9	Response to osmotic stress	8.11E-05
CSRP3	Cardiac muscle tissue development	5.95E-05
CHD2	Chromodomain helicase DNA binding protein 2	5.62E-05
TTC7B	Binding	4.93E-05
TADA2B	Regulation of transcription	3.82E-05
Gga.52217	Homophilic cell adhesion	1.55E-05
RSAD2	Radical S-adenosyl methionine domain containing 2	1.17E-05
FAM5B	Nervous system development	7.98E-06
ZNF639	Transcription factor activity	3.82E-06
SYN1	Synapsin I	3.63E-06
OCIAD1	Endosome	3.12E-06
TMC5	Integral to membrane	1.80E-06
GFPT2	Glutamine-fructose-6-phosphate transaminase 2	2.22E-07
ADRBK1	Beta-adrenergic receptor kinase 1	1.68E-07
ZNFX1	Metal ion binding	1.67E-07
DTX3L	Deltex 3-like	1.88E-08
EZH2	Chromatin modification	1.57E-08

Likelihood ratio tests based on a branch site model were performed on the CDS alignments of orthologs among chickens, zebra finches, turkeys, and ground tits. The *P *value was calculated and then adjusted with a conservative correction for multiple testing (false discovery rate <0.05, Holm's correction).

Among the positively selected genes, we found two genes (ADRBK1 and HSD17B7) that may be involved in the adrenaline response and steroid hormone biosynthesis. ADRBK1 is a ubiquitous cytosolic enzyme that specifically phosphorylates the activated form of the beta-adrenergic and related G-protein-coupled receptors. HSD17B7 is an enzyme that can oxidize or reduce estrogens and androgens in mammals, thereby regulating the biological potency of these steroids [27].

Interestingly, two genes encoding key proteins in signal transduction were found to be under positive selection: one encodes the receptor TACR1 for tachykinin/substance P. The binding of substance P to TACR1 has been associated with the transmission of stress signals and pain, the contraction of smooth muscles, and inflammation [28]. The other gene encodes GFPT2, which catalyzes the rate-limiting step in the formation of hexosamine, a precursor for N-linked and O-linked glycosylation reactions. These reactions modify proteins and lipids before their participation in signal transduction, trafficking, or secretion and other processes [29].

## Discussion

The ground tit genome sequence described here represents a high quality avian genome sequenced by a next generation sequencing platform. Its assembly is comparable to other available genomes in terms of genome coverage, and has a better sequence continuity. Furthermore, the high sequencing depth confirmed the accuracy of the assembly at the nucleotide level. Overall, the high quality of this genome makes it a valuable resource for comparative genomics. Our micro-synteny analysis among the ground tit, zebra finch, and chicken illustrated that their genome structures were relatively conserved. Our observation agrees with the reports of conserved overall synteny between zebra finch and chicken [30] and also between chicken and turkey [31], indicating a conserved genome structure among these avian species. However, this inference requires further confirmation using more sequenced avian genomes.

The phylogeny of the ground tit has been controversial. It was classified as a species of the family Corvidae, and this classification is reflected in its other common name, Hume's groundpecker. However, some reports have suggested that in genetic distance it is closer to the family Paridae [7], according to independent datasets drawn from comparative osteology, the nuclear c-myc gene, and the mitochondrial cytochrome *b *gene. Our phylogenetic analysis confirmed that the ground tit does not belong to the Corvidae family, agreeing with the previous report that analyzed certain nuclear genes [7]. The species tree presented here, based on the current available genomic data, provided convincing evidence that the ground tit is not a crow species; however more genomes belonging to Paridae and its relatives are required to confirm the ground tit's taxonomic status and evolutionary relationships.

The evolution of hormone-behavior adaptations may have helped the ground tit cope with the extreme environments on the QTP. This kind of adaptation strategy, involving hormone regulatory mechanisms for physiology and behavior in extreme conditions, has been used by birds that breed on the arctic tundra [32,33]. We identified several genes that are under positive selection, including ADRBK1 and HSD17B7, which are involved in the adrenaline response and steroid hormone biosynthesis, correlating with an adaptation strategy of hormone regulatory mechanisms for physiology and behavior in extreme conditions. Further, both ADRBK1 and CSRP3 play significant roles in cardiac muscle contraction and heart development. Neuropeptides and glycosylation modification may be linked to adaptation strategies, but this hypothesis requires further support. Mutations in the CSRP3 gene have been suggested to cause heritable forms of hypertrophic cardiomyopathy and dilated cardiomyopathy in humans [34]. Nevertheless, unlike other QTP animals (for example, the yak), we did not find evidence of positive selection for genes associated with energy metabolism in the ground tit. A previous study observed some genetic adaptation to the highland environment in the Tibetan population [2]; however, considering the relatively short time that humans have lived in this region and the different energy requirements of mammals and birds, the genetic mechanism of adaptation in the ground tit may be different from that in mammals, for which the hypoxia-inducible factors pathway may play an important role, and which calls for a wider genomic comparison of high land species.

## Conclusions

The ground tit genome was sequenced and compared with the genomes of other animals, especially birds. Phylogeny analysis confirmed that the ground tit did not belong to the Corvidae family. The roles of genes related to cardiac function were implicated in the adaptation of ground tit to the extreme highland environment.

## Methods

### Source of samples

A male adult ground tit from the Qinghai-Tibetan Plateau was used in this study. Genomic DNA was collected from the peripheral blood cells of this individual.

### Sample preparation and sequencing

We constructed seven paired-end libraries, with sizes ranging from 200 bp to 20 kb from the genomic DNA of the ground tit. The libraries were prepared following the manufacturer's standard instructions and sequenced on an Illumina Hiseq platform. Whole genome sequencing was done as described previously [11]. A total of 184.5 Gb of data were generated from these libraries.

### Genome assembly

Before assembly, a series of filtering steps was undertaken to filter the low-quality sequencing reads; 119.0 Gb (or 108.2 folds) data were retained for assembly. The sequences were assembled *de novo *by the *de Bruijn *graph-based assembler SOAPdenovo [10]. The reads from the short insert libraries (<2 kb) were first used to build the contigs, and then all the paired-end reads were realigned onto the contig sequences to construct the scaffolds. We then determined the extent of the shared paired-end relationships between each pair of contigs, weighted the rate of consistent and conflicting paired ends and constructed the scaffolds step by step, in increasing order of insert size. Finally, we used the paired-end information to retrieve read pairs (that had one end mapped to a unique contig and the other located in a gap region) and performed a local assembly for these collected reads to fill the gaps. The genome assembly statistics are shown in Table S2.

The *Pseudopodoces humilis *whole-genome shotgun project has been deposited in the DDBJ/EMBL/GenBank databases under the project accession ANZD00000000. Correspondence and requests for materials should be addressed to Jun Wang (wangj@genomics.org.cn).

### Detection of single nucleotide polymorphisms

To identify single nucleotide polymorphisms of ground tit, we mapped all the high-quality reads from the short insert libraries (<2 kb) onto the ground tit assembly using SOAPaligner in the gap-free mode and allowing three mismatches. SOAPsnp was used to call the single nucleotide variations. After quality control and filtering, 1.7 M single nucleotide polymorphisms were identified.

### Annotation of protein-coding genes

We used homology, *ab-initio *prediction, and ESTs (zebra finch and chicken) to identify protein-coding genes, and then built a consensus gene set that contained all the predicted genes. For the homology-based gene prediction, we aligned zebra finch, chicken, lizard, and human protein sequences (Ensembl release 66) to the ground tit genome using TBLASTN, genBlastA [35], and Genewise [36]. We then aligned the ESTs to the assembled ground tit genome sequences using BLAT [37] and generated EST hints for AUGUSTUS [38]. We used repeat-masked ground tit genome sequences for the *ab-initio *prediction. We used AUGUSTUS to predict protein-coding genes with parameters that were trained from a set of high quality homologous prediction proteins, given EST and homolog hints as extrinsic evidence. We then used Genscan [39] with the human parameter file to predict protein-coding genes.

The Ensembl method was used to merge all the gene predictions from the various sources as follows. We picked homologs in four layers of increasing evolutionary distance: zebra finch, chicken, lizard, and human. The sequence that was most like the query protein in each layer was picked and added to the final gene set. Single-exon genes that were derived from retrotransposition and contained a frame error were filtered out. We also removed multi-exon genes that were not supported by whole genome synteny: ≥3 frame errors were required; while multi-exon genes that were supported by synteny were only removed if they contained ≥8 frame errors. For the *ab-initio *prediction set, partial genes and small genes that had coding lengths <150 bp were filtered out. We then aligned the predictions to a transposable element (TE) protein database [40] using BlastP with an E-value cut-off of 1e-5 and genes that aligned to transposable elements at >50% were removed from the final gene set. Next, the *ab-initio *predicted genes were aligned to the SwissProt/TrEMBL [41] database and those showed >30% sequence similarity were retained. When gene sequences overlapped, the sequence with the longest length aligning to the database was chosen so that no gene was represented twice in the final dataset.

We annotated the genes in the final dataset using InterProScan [42] to assign Pfam, PRINTS, PROSITE, ProDom, SMART, and PANTHER motifs and domains to the sequences. GO annotations were retrieved from the results of the InterProScan. We also mapped the final gene set to KEGG pathway maps by searching the KEGG databases for the best hit for each gene.

### Identification of repeat sequences

We first identified known transposable elements using RepeatMasker [43] against the Repbase [40] TE library (version 2011-09-20), and then executed RepeatProteinMask [43]. We constructed a *de-novo *ground tit repeat library using RepeatModeler [44]. RepeatMasker was run on the genome sequences, using the RepeatModeler consensus sequence as the library. We predicted tandem repeats using tandem repeats finder (TRF) [45].

### Construction of gene families

The protein-coding genes from six species (human, mouse, lizard, chicken, zebra finch, and turkey) were downloaded from Ensembl [46] release 66; duck protein-coding genes were obtained from pre.ensembl.org, and crow protein-coding genes were from the Beijing Genomics Institute. We used the tree-building method used in TreeFam [47] to define gene families. *H. sapiens *and *M. musculus *sequences were used as the outgroup. Genes that were predicted to encode <30 amino acids were filtered out. For genes with alternative splicing variants, the longest transcript was selected to represent the gene. A total of 5,212 single-copy families, including the ground tit final gene set and the eight orthologous species, were used to reconstruct phylogenies and estimate divergence time. The four-fold-degenerate sites were extracted from each family and concatenated to one supergene for each species. jModeltest [48] was used to select the best substitution model (GTR+gamma+I) and MrBayes [49] was used to reconstruct the phylogenetic tree. The program Mcmctree implemented in the PAML [50] package was used to estimate the divergence time. The calibration time was achieved from the Date-A Clade and the Fossil Record website [51]. We used the Café program [52] to identify gene families that had undergone expansion and contraction.

### Calculation of synonymous (dS) and non-synonymous (dN) nucleotide substitutions

A total of 8,136 single-copy gene families for *T. guttata*, *G. gallus*, *M. gallopavo *and *P. humilis *were defined using the TreeFam method. We applied the Guidance software program [53] to filter out unreliable alignment regions. We used the Yang-Nielsen model [54] to calculate dN and dS values for each pair of *ground tit *and *T. guttata *genes. To estimate lineage-specific dN and dS values, the alignment that remained after the Guidance filtering was used to calculate in-branch dN and dS values using the codeml program of the PAML [50] package with the F3×4 model, and to calculate different ω ratios across branches and a single ω ratio across sites, and a separate estimation of κ per gene and a given tree topology.

### Three-way avian genome alignment

Multiple (three-way) alignments were built on the ground tit, zebra finch, and chicken genomes using Multiz and following the topology of species trees. The chicken genome was set as the reference and the input pairwise alignments (chicken *vs*. ground tit, chicken *vs*. zebra finch, zebra finch *vs*. ground tit) were obtained using Lastz, with the following parameters: O = 600, T = 2, E = 150, H = 0, Y = 15,000, L = 3,000, and K = 4,500. The raw alignments were processed using the China/Net package [55]. The ground tit genome was masked with RepeatMasker with the '-s' option and TRF tandem repeats of period ≤12. The zebra finch and chicken repeat-masked genomes were downloaded from UCSC [56].

### Detection genes under positive selection

To identify genes under positive nature selection during evolutionary history, we constructed 1:1 orthologs among ground tit, chicken, zebra finch, and turkey. To reduce the bias of the gene annotations for these birds, we selected the zebra finch transcripts as the reference sequences and mapped them to each of the other three species via syntenic alignments. Details of the procedure are as follows: (1) Syntenic alignment. The latest chicken (galGal4), zebra finch (taeGut1), and turkey (melGal1) genome sequences were obtained from UCSC and the syntenic pairwise whole-genome alignments between zebra finch and each of the other three species were built using Lastz and the China/Net package; (2) Ortholog identification. The transcripts of zebra finch were mapped to the genomes of the other three species according to the syntenic alignment and some criteria ((a) the mapped regions covered ≥80% of the coding region; (b) ≤10% of the coding region was in sequence gaps or low quality sequence; (c) no frame-shift indels unless they were compensated for within 15 bases; and (d) no in-frame stop codons and all splice sites were conserved). The final orthologous sets were obtained by selecting the longest transcript mapped to the species for each gene and each ortholog should contain at least the zebra finch and ground tit sequences; (3) Species-tree reconstruction. Each gene-tree should be reconstructed via the species above because of the variable number of genes for each ortholog; (4) Likelihood Ratio Test (LRT). The ratios of non-synonymous substitutions per non-synonymous site (dN) to synonymous substitutions per substitution site (dS), indicated by dN/dS (also termed w), were estimated for each gene from the coding sequence alignment of each of the identified orthologous groups by the maximum likelihood (ML) with the codeml program from PAML4 [50]. Two models were implemented to test the statistical significance of selective pressure specifically on the ground tit branch; one was the one-ratio model that acts as the null model (NSsites = 0, model = 0), and the other was model 2 (NSsites = 2). The two models were compared with the LRT, calculated from the log likelihood (lnL) values for both models. The *P *values were obtained by calculating twice the difference between lnL_model2 _and lnL_one-ratio _and compared with a chi-square distribution.

### Rapidly and slowly evolving GO function categories

To identify GO function categories under rapid or slow evolution, the GO annotations of the zebra finch genome were downloaded from the Ensembl database. Only the GO categories that contained at least 20 genes were retained for further analysis. Orthologs of ground tit and zebra finch were selected. First, we calculated the average *k_a _*and *k_s _*values for genes annotated to a given GO term as

$$k_{a}=\frac{\Sigma_{i\in T}a_{i}}{\Sigma_{i\in T}A_{i}},\;ks=\frac{\Sigma_{i\in T^{S_{i}}}}{\Sigma_{i\in T}S_{i}}$$

where *a_i _*and *A_i _*are the numbers of non-synonymous substitutions and sites, and *s_i _*and *S_i _*are the numbers of synonymous substitutions and sites in gene *i*, as estimated by PAML, respectively.

The expected proportion of non-synonymous substitutions *P_A _*in a GO category *C *was then estimated as:

$$P_{A}=\frac{k_{a}\sum_{i\in C}A_{i}}{k_{a}\sum_{i\in C}A_{i}+k_{s}\sum_{i\in C}S_{i}}$$

Then, for a given GO category C, we used a binomial distribution to estimate the divergence of the proportion of non-synonymous substitutions and synonymous sites between the observed and the expected as:

$$P_{c}=\sum_{j=a_{c}}^{a_{c}+s_{c}}\left(\begin{array}{c} a_{c}+s_{c}\\ j \end{array}\right)P_{A}^{j}{\left(1-P_{A}\right)}^{a_{c}+s_{c}-j}$$

where *a_C _*and *s_C _*are the total number of non-synonymous and synonymous substitutions in GO category *C*, respectively.

Rapidly (or slowly) evolving categories were detected by calculating the probability that a category contains equal or more (or less) non-synonymous substitutions, conditional on the total number of observed substitutions.

To determine whether a subset of the categories was evolving under significantly high (low) constraints, we first computed the number of GO categories with P_C _values less than a given threshold value (0.05, 0.01, 0.001, 0.0001). We then repeated this procedure 10,000 times on the same dataset after randomly permuting the GO annotation (all GO categories assigned to a specific gene were kept together to preserve the hierarchical structure of the GO categories). Finally, we tested the null hypothesis that the number of biologically meaningful categories with P_C _values below the chosen threshold was no more than the expected values from the randomly composed categories by counting how many of the latter had lower P_C _values. A rejection of this null hypothesis implied that the level of constraint was significantly higher (lower) than average in some biologically meaningful categories. The average number of categories in the randomized datasets with P_C _values below the threshold was the expected number of false-positives among the putatively rapidly (slowly) evolving categories.

## Competing interests

The authors declare that they have no competing interests.

## Authors' contributions

JW and YL designed the project. XQ, YL, JX, JL, LL, ZW, JZ, YC, and CG prepared the DNA samples and generated the sequencing data. YL, RH, and QC performed the genome assembly and annotation. YH, MH, SW, XZ, and RH performed the comparative genomics and evolution analysis. QC, YY, and SP wrote the paper. All authors read and approved the final manuscript.

## Supplementary Material

Additional file 1 **Tables S1-S13 and Figures S1-S7**. **Table S1**. Summary of the sequencing data of *P. humilis*. **Table S2**. Statistics of ground tit genome assembly. **Table S3**. Statistics of transposable elements detected in the ground tit assembly. **Table S4**. Comparison of transposable elements among chicken, zebra finch, and ground tit. **Table S5**. General statistics of each gene set and integrated predictions. **Table S6**. Genes annotated via functional databases. **Table S7**. Statistics of ncRNA prediction in the assembly. **Tables S8-S10**. Functional analysis of gene families of expansion and contraction in ground tit. **Tables S11-S13**. Analysis of rapidly and slowly evolving categories. **Figure S1**. Local GC content distribution of the *P. humilis*, chicken, zebra finch, and human genomes. **Figure S2**. Comparison of gene parameters between ground tit and chicken, zebra finch, and human. **Figure S3**. Supporting evidence for gene models. **Figure S4**. Distribution of orthologous protein identities between chicken and other species for a subset of strictly conserved single-copy orthologs. **Figure S5**. Micro-synteny between genomes of chicken and ground tit. **Figure S6**. Venn diagram showing the amount of sequence (in Mbp) aligned among the three avian genomes. **Figure S7**. Dynamic evolution of orthologous gene clusters. Click here for file
