# Correction: The genome sequence of the ground tit *Pseudopodoces humilis* provides insights into its adaptation to high altitude

**DOI:** 10.1186/gb-2014-15-2-r33

**Published:** 2014-02-14

**Authors:** Qingle Cai, Xiaoju Qian, Yongshan Lang, Yadan Luo, Jiaohui Xu, Shengkai Pan, Yuanyuan Hui, Caiyun Gou, Yue Cai, Meirong Hao, Jinyang Zhao, Songbo Wang, Zhaobao Wang, Xinming Zhang, Rongjun He, Jinchao Liu, Longhai Luo, Yingrui Li, Jun Wang

**Affiliations:** 1 BGI-Shenzhen, Beishan Industrial Zone, Yantian District, Shenzhen 518083, China; 2 Department of Biology, University of Copenhagen, DK-1165 Copenhagen, Denmark; 3 King Abdulaziz University, Abdulla Alsulaiman Road, Jeddah 21589, Saudi Arabia

## Correction

During preparation of our article [1] for final publication, we introduced a few minor changes to the text of the article; additionally, one of the authors was removed from the list of authors and one factual error was corrected. These changes are listed in detail below.

1. Fumin Lei is no longer listed as an author of this article. Instead, his helpful input is noted in the acknowledgements section.

2. The provisional version of this article mistakenly stated that zebra finch belongs to the Paridae family. We have now corrected this error to reflect the classification of this species to the Estrildidae family.

3. In the abstract of the provisional version of the article we stated that the phylogeny of the ground tit was confirmed as belonging to the Paridae family. We have now re-phrased this sentence to say that ground tit phylogeny was confirmed as not belonging to the Corvidae family.

4. In the conclusions of the provisional version of the article we stated that the phylogeny of the ground tit was confirmed as not belonging to the Corvidae family but to the Paridae family. We have now re-phrased this conclusion to say that ground tit phylogeny was confirmed as not belonging to the Corvidae family.
