# Supplementary material for: Genome sequence of ground tit Pseudopodoces humilis and its adaptation to high altitude
Source: Genome Biol. 2013 Mar 28;14(3):R29. doi: 10.1186/gb-2013-14-3-r29 (PMC4053790; doi:10.1186/gb-2013-14-3-r29)
Supplement: Additional file 1 — Tables S1-S13 and Figures S1-S7. Table S1. Summary of the sequencing data of P. humilis. Table S2. Statistics of ground tit genome assembly. Table S3. Statistics of transposable elements detected in the ground tit assembly. Table S4. Comparison of transposable elements among chicken, zebra finch, and ground tit. Table S5. General statistics of each gene set and integrated predictions. Table S6. Genes annotated via functional databases. Table S7. Statistics of ncRNA prediction in the assembly. Tables S8-S10. Functional analysis of gene families of expansion and contraction in ground tit. Tables S11-S13. Analysis of rapidly and slowly evolving categories. Figure S1. Local GC content distribution of the P. humilis, chicken, zebra finch, and human genomes. Figure S2. Comparison of gene parameters between ground tit and chicken, zebra finch, and human. Figure S3. Supporting evidence for gene models. Figure S4. Distribution of orthologous protein identities between chicken and other species for a subset of strictly conserved single-copy orthologs. Figure S5. Micro-synteny between genomes of chicken and ground tit. Figure S6. Venn diagram showing the amount of sequence (in Mbp) aligned among the three avian genomes. Figure S7. Dynamic evolution of orthologous gene clusters. [file gb-2013-14-3-r29-S1.DOCX]

Table S1 Summary of sequencing data of ground tit (P.humilis, the same below).

| **Pair-end Libraries** | **Insert Size** | **Ave Reads Length(bp)** | **Raw Data(Gb)** | **Sequence Depth(X)** | **Clean Data(Gb)** | | **Sequence Depth(X)** |
| --- | --- | --- | --- | --- | --- | --- | --- |
| Solexa  Reads | 170bp | 100 | 29.4 | 26.7 | 27.3 | 24.9 | |
|  | 500bp | 100 | 36.6 | 33.3 | 32.8 | 29.8 | |
|  | 800bp | 100 | 25.2 | 22.9 | 22.1 | 20.1 | |
|  | 2Kb | 49 | 38.8 | 35.3 | 25.3 | 23.0 | |
|  | 5Kb | 49 | 17.7 | 16.1 | 6.0 | 5.4 | |
|  | 10Kb | 49 | 18.0 | 16.3 | 3.2 | 2.9 | |
|  | 20Kb | 49 | 18.9 | 17.2 | 2.4 | 2.2 | |
| Total |  |  | 184.5 | 167.7 | 119.0 | 108.2 | |

*Sequence depth was calculated in terms of the estimated genome size as 1.1Gb

Table S2 Statistic of ground tit genome assembly

|  | **Contig** | | **Scaffold** | |
| --- | --- | --- | --- | --- |
|  | **Size(bp)** | **Number** | **Size(bp)** | **Number** |
| N90 | 36,106 | 6,710 | 1,794,782 | 97 |
| N80 | 67,948 | 4,670 | 3,924,218 | 56 |
| N70 | 98,806 | 3,420 | 7,025,951 | 36 |
| N60 | 130,064 | 2,510 | 11,543,148 | 24 |
| N50 | 164,707 | 1,802 | 16,337,386 | 16 |
| Longest | 1,467,724 |  | 77,483,305 |  |
| Total Size | 1,032,567,648 |  | 1,045,634,407 |  |
| Total Number(>=100bp) |  | 47,542 |  | 26,033 |
| Total Number(>=2Kb) |  | 14,539 |  | 1,027 |

Table S3 Statistic of transpose elements detected in the ground tit assembly.

|  | **Repbase TEs** | | **TE protiens** | | **De novo** | | **Combined TEs** | |
| --- | --- | --- | --- | --- | --- | --- | --- | --- |
| **Type** | **Length (bp)** | **%^a^** | **Length (bp)** | **%** | **Length (bp)** | **%** | **Length (bp)** | **%** |
| **DNA** | 3,531,906 | 0.34 | 133,672 | 0.01 | 522,901 | 0.05 | 3,853,875 | 0.37 |
| **LINE** | 35,835,383 | 3.43 | 19,253,982 | 1.84 | 32,843,491 | 3.14 | 38,682,776 | 3.7 |
| **SINE** | 831,512 | 0.08 | 0 | 0 | 301,720 | 0.03 | 952,164 | 0.09 |
| **LTR** | 18,609,953 | 1.78 | 5,078,328 | 0.49 | 19,368,798 | 1.85 | 25,425,341 | 2.43 |
| **Other** | 8,936 | 0 | 0 | 0 | 0 | 0 | 8,936 | 0 |
| **Un^b^** | 457,676 | 0.04 | 0 | 0 | 4,608,107 | 0.44 | 5,065,472 | 0.48 |
| **Total** | 58,653,007 | 5.61 | 24,465,077 | 2.34 | 57,529,268 | 5.5 | 72,652,236 | 6.95 |

*Repbase TEs : Transpose elements identified using Repeatmasker with in Repbase as the library;TE proteins : Transpose elements identified using RepeatProteinMasker with the transposons protein in the Repbase as the library; De novo: Transpose elements identified using RepeatMasker with the library constructed by Repeatmodeler.

*^a^ % : Percent of genome.

*^b^Un: Unclassified into any types of TE.

Table S4 Comparison of TE among chicken, zebra finch and ground tit

|  | **zebra finch** | | **chicken** | | **ground tit** | |
| --- | --- | --- | --- | --- | --- | --- |
| **Type** | **Length (bp)** | **% in genome** | **Length**  **(bp)** | **% in genome** | **Length (bp)** | **% in genome** |
| **DNA** | 3,254,442 | 0.26 | 10,839,260 | 0.98 | 3,853,875 | 0.37 |
| **LINE** | 42,251,706 | 3.43 | 72,076,279 | 6.50 | 38,682,776 | 3.70 |
| **LTR** | 45,647,421 | 3.70 | 17,634,348 | 1.59 | 952,164 | 0.09 |
| **SINE** | 937,679 | 0.08 | 701,840 | 0.06 | 25,425,341 | 2.43 |
| **Other** | 7,972 | 0.00 | 2,563 | 0.00 | 8,936 | 0.00 |
| **Unknown** | 488,742 | 0.04 | 480,549 | 0.04 | 5,065,472 | 0.48 |
| **Total** | 92,587,962 | 7.51 | 101,734,839 | 9.18 | 72,652,236 | 6.95 |

Table S5 General statistics of each gene set and integrated prediction.

| **Gene Set** | | **Number** | **Average transcript length (bp)** | **Average CDS length (bp)** | **Average exon per gene** | **Average exon length (bp)** | **Average intron length (bp)** |
| --- | --- | --- | --- | --- | --- | --- | --- |
| ***De novo*** | *AUGUSTUS* | 20032 | 19780 3/8 | 1455 3/7 | 8 2/3 | 168 1/2 | 2398 2/3 |
|  | *GENSCAN* | 34402 | 22264 1/3 | 1408 1/2 | 8 | 174 2/5 | 2947 5/9 |
| **Homolog** | *T.* *guttata* | 15,630 | 21,223 | 1,432 | 9 | 158 | 2,451 |
|  | *G.* *gallus* | 14,833 | 22,542 | 1,556 | 10 | 156 | 2,343 |
|  | *A.carolinensis* | 14,660 | 18,340 | 1,549 | 10 | 158 | 1,908 |
|  | *H.* *sapiens* | 12,817 | 31,052 | 1,772 | 12 | 152 | 2,757 |
| **EST** | *T.* *guttata* | 52,604 | 92,088 | 690 | 4 | 156 | 26,606 |
|  | *G.* *gallus* | 69,255 | 14,038 | 1,128 | 8 | 139 | 1,813 |
| **Final** | | 16,998 | 20,192 | 1,402 | 9 | 158 | 2,395 |

*Denovo : Gene sets were obtained through de novo prediction methods using the software *AUGUSTUS* and *GENSCAN.* Homolog: Gene sets were obtained through aligning homolog protein from the four species (*T.* *guttata, G.* *gallus, A.carolinensis*

and *H.* *sapiens*) to the ground tit assembly.

EST: Gene structures were identified by mapping the EST sequences from *T.* *guttata and G.* *gallus* to the assembly

Table S6 Gene annotated via functional databases

|  | Database | Number | Percent(%) |
| --- | --- | --- | --- |
| Annotated | InterPro | 15,025 | 88.39 |
|  | GO | 11,649 | 68.53 |
|  | KEGG | 12,496 | 73.51 |
|  | Swissprot | 15,796 | 92.93 |
|  | TrEMBL | 16,732 | 98.44 |
| Unannotated | | 180 | 1.06 |

Table S7 Statistic of ncRNA prediction in the assembly

| **Tpye** | | **Copy** | **Aver_Length(bp)** | **Total_length(bp)** |
| --- | --- | --- | --- | --- |
| miRNA | | 193 | 82.18 | 15,861 |
| tRNA | | 116 | 76.03 | 8,820 |
| **rRNA** | 18S rRNA | 1 | 73 | 73 |
|  | 28S rRNA | 7 | 87.71 | 614 |
|  | Total rRNA | 8 | 85.88 | 687 |
| **snRNA** | CD-box snRNA | 119 | 92.35 | 10,990 |
|  | HACA-box snRNA | 73 | 140.71 | 10,272 |
|  | scaRNA snRNA | 14 | 166.14 | 2,326 |
|  | snRNA snRNA | 1 | 62 | 62 |
|  | splicing snRNA | 32 | 140.31 | 4,490 |
|  | Total snRNA | 239 | 117.74 | 28,140 |

Table S8 KEGG pathways enrichment analysis of gene family expansion of ground tit

| **MapID** | **MapTitle** | **Pvalue** | **Adjusted Pvalue** |
| --- | --- | --- | --- |
| map05322 | Systemic lupus erythematosus | 5.34E-19 | 7.63E-17 |
| map05414 | Dilated cardiomyopathy | 2.02E-14 | 1.44E-12 |
| map05310 | Asthma | 1.32E-11 | 6.30E-10 |
| map05143 | African trypanosomiasis | 3.26E-08 | 1.17E-06 |
| map05330 | Allograft rejection | 1.33E-07 | 3.79E-06 |
| map05416 | Viral myocarditis | 3.06E-07 | 7.29E-06 |
| map05320 | Autoimmune thyroid disease | 4.81E-07 | 9.83E-06 |
| map05146 | Amoebiasis | 5.70E-07 | 1.02E-05 |
| map05340 | Primary immunodeficiency | 9.00E-07 | 1.30E-05 |
| map04640 | Hematopoietic cell lineage | 9.09E-07 | 1.30E-05 |
| map04672 | Intestinal immune network for IgA production | 1.20E-06 | 1.56E-05 |
| map05140 | Leishmaniasis | 3.07E-06 | 3.66E-05 |
| map04662 | B cell receptor signaling pathway | 4.96E-06 | 5.46E-05 |
| map00100 | Steroid biosynthesis | 2.09E-05 | 2.03E-04 |
| map05150 | Staphylococcus aureus infection | 2.12E-05 | 2.03E-04 |
| map04145 | Phagosome | 5.54E-05 | 4.95E-04 |
| map04664 | Fc epsilon RI signaling pathway | 2.27E-04 | 1.91E-03 |
| map04666 | Fc gamma R-mediated phagocytosis | 3.17E-04 | 2.52E-03 |
| map05410 | Hypertrophic cardiomyopathy (HCM) | 4.42E-04 | 3.33E-03 |
| map05130 | Pathogenic Escherichia coli infection | 6.31E-04 | 4.51E-03 |
| map05162 | Measles | 7.48E-04 | 5.10E-03 |
| map04650 | Natural killer cell mediated cytotoxicity | 9.72E-04 | 6.12E-03 |
| map05323 | Rheumatoid arthritis | 9.84E-04 | 6.12E-03 |
| map00524 | Butirosin and neomycin biosynthesis | 1.29E-03 | 7.35E-03 |
| map00965 | Betalain biosynthesis | 1.29E-03 | 7.35E-03 |
| map00982 | Drug metabolism - cytochrome P450 | 1.75E-03 | 9.62E-03 |
| map04610 | Complement and coagulation cascades | 1.96E-03 | 1.04E-02 |
| map04080 | Neuroactive ligand-receptor interaction | 2.16E-03 | 1.10E-02 |
| map00052 | Galactose metabolism | 2.96E-03 | 1.46E-02 |
| map04710 | Circadian rhythm - mammal | 3.80E-03 | 1.81E-02 |
| map00140 | Steroid hormone biosynthesis | 4.35E-03 | 2.00E-02 |
| map00120 | Primary bile acid biosynthesis | 6.85E-03 | 3.06E-02 |
| map00500 | Starch and sucrose metabolism | 7.96E-03 | 3.45E-02 |
| map01040 | Biosynthesis of unsaturated fatty acids | 8.29E-03 | 3.49E-02 |
| map04020 | Calcium signaling pathway | 9.86E-03 | 4.03E-02 |

Table S9 GO enrichment analysis of significantly contraction of gene families in ground tit.

| **GO_ID** | **GO_Term** | **GO_Class** | **Pvalue** | **Adjusted Pvalue** |
| --- | --- | --- | --- | --- |
| GO:0005524 | ATP binding | MF | 1.03E-23 | 2.27E-22 |
| GO:0004672 | protein kinase activity | MF | 1.95E-23 | 3.07E-22 |
| GO:0006468 | protein phosphorylation | BP | 2.90E-23 | 3.65E-22 |
| GO:0004674 | protein serine/threonine kinase activity | MF | 1.64E-22 | 1.48E-21 |
| GO:0003824 | catalytic activity | MF | 1.60E-09 | 4.03E-09 |
| GO:0016459 | myosin complex | CC | 3.57E-07 | 8.04E-07 |
| GO:0003774 | motor activity | MF | 9.88E-06 | 2.01E-05 |
| GO:0005488 | binding | MF | 3.12E-05 | 6.14E-05 |

Table S10 KEGG pathways enrichment analysis of gene family contraction of ground tit

| **MapID** | **MapTitle** | **Pvalue** | **Adjusted Pvalue** |
| --- | --- | --- | --- |
| map05221 | Acute myeloid leukemia | 9.72E-14 | 1.20E-12 |
| map05211 | Renal cell carcinoma | 1.84E-13 | 1.20E-12 |
| map04012 | ErbB signaling pathway | 8.48E-12 | 3.67E-11 |
| map04660 | T cell receptor signaling pathway | 3.64E-11 | 1.18E-10 |
| map04630 | Jak-STAT signaling pathway | 1.14E-10 | 2.95E-10 |
| map04510 | Focal adhesion | 1.40E-09 | 3.03E-09 |
| map04360 | Axon guidance | 4.95E-09 | 9.19E-09 |
| map04810 | Regulation of actin cytoskeleton | 4.51E-08 | 7.32E-08 |
| map05416 | Viral myocarditis | 6.42E-05 | 9.27E-05 |
| map04530 | Tight junction | 1.18E-03 | 1.54E-03 |

Table S11 Identification of rapidly and slowly evolving categories

|  | **Higher Ka/Ks** | | | | **Lower Ka/Ks** | | | |
| --- | --- | --- | --- | --- | --- | --- | --- | --- |
| **Threshold** | 0.05 | 0.01 | 0.001 | 0.0001 | 0.05 | 0.01 | 0.001 | 0.0001 |
| **Observed^a^** | 39 | 35 | 25 | 22 | 74 | 69 | 58 | 55 |
| **Expected^b^** | 35.9 | 27.3 | 19.4 | 14.1 | 45.1 | 35.1 | 25.4 | 18.8 |
| **p-value^c^** | 0.3346 | 0.1076 | 0.1552 | 0.0556 | 0 | 0 | 0 | 0 |

^a^ number of significant categories; ^b^ average number of significant categories identified in 1,000 random sets; ^c^ proportion of random sets which have as many or more categories as observed in the data set

Table S12 Function analysis of rapidly evolving GO categories

| **#GO ID** | **Gene number** | **GO name** | **GO Class** | **dN/dS** | **Amino Acid divergence** | **pvalue** |
| --- | --- | --- | --- | --- | --- | --- |
| GO0006281 | 30 | DNA repair | BP | 0.216529 | 0.045323147 | 6.60E-43 |
| GO0006955 | 29 | immune response | BP | 0.264123 | 0.058038606 | 1.55E-38 |
| GO0005488 | 65 | binding | MF | 0.168479 | 0.038970184 | 5.00E-36 |
| GO0055114 | 180 | oxidation-reduction process | BP | 0.155607 | 0.034807421 | 1.76E-30 |
| GO0005576 | 119 | extracellular region | CC | 0.146102 | 0.037863877 | 1.36E-24 |
| GO0016491 | 130 | oxidoreductase activity | MF | 0.155183 | 0.034252 | 7.97E-20 |
| GO0008168 | 25 | methyltransferase activity | MF | 0.196468 | 0.044695952 | 2.12E-17 |

Table S13 Function analysis of slowly evolving GO categories

| **#GO ID** | **gene number** | **GO name** | **GO Class** | **dN/dS** | **Amino Acid divergence** | **pvalue** |
| --- | --- | --- | --- | --- | --- | --- |
| GO0006811 | 88 | ion transport | BP | 0.055343 | 0.011889018 | 2.3E-114 |
| GO0005216 | 69 | ion channel activity | MF | 0.0511536 | 0.011135012 | 4.5E-110 |
| GO0016020 | 654 | membrane | CC | 0.0938611 | 0.021579844 | 1.79E-73 |
| GO0006816 | 20 | calcium ion transport | BP | 0.0453635 | 0.010584069 | 3.30E-54 |
| GO0006468 | 264 | protein phosphorylation | BP | 0.0918571 | 0.018923426 | 6.67E-51 |
| GO0016881 | 37 | acid-amino acid ligase activity | MF | 0.0592329 | 0.011272748 | 6.10E-48 |
| GO0004672 | 260 | protein kinase activity | MF | 0.0932743 | 0.019198262 | 1.02E-44 |
| GO0004674 | 262 | protein serine/threonine kinase activity | MF | 0.0943936 | 0.019507182 | 3.81E-43 |
| GO0007275 | 45 | multicellular organismal development | BP | 0.0384926 | 0.012668574 | 2.91E-38 |
| GO0007169 | 26 | transmembrane receptor protein tyrosine kinase signaling pathway | BP | 0.0569002 | 0.014304341 | 7.29E-35 |
| GO0051056 | 24 | regulation of small GTPase mediated signal transduction | BP | 0.0695871 | 0.015284919 | 2.76E-33 |
| GO0006813 | 48 | potassium ion transport | BP | 0.0579675 | 0.01238364 | 2.02E-31 |
| GO0023034 | 103 | intracellular signaling pathway | BP | 0.0880874 | 0.018538928 | 9.00E-29 |
| GO0006355 | 257 | regulation of transcription, DNA-dependent | BP | 0.0899119 | 0.017517482 | 4.66E-28 |
| GO0005856 | 48 | cytoskeleton | CC | 0.0781367 | 0.016828868 | 1.44E-26 |
| GO0004871 | 64 | signal transducer activity | MF | 0.0701099 | 0.01605921 | 4.11E-24 |
| GO0005887 | 38 | integral to plasma membrane | CC | 0.0683148 | 0.016563738 | 3.73E-22 |
| GO0007165 | 222 | signal transduction | BP | 0.0944549 | 0.020904661 | 7.08E-22 |
| GO0008565 | 21 | protein transporter activity | MF | 0.0551824 | 0.011515741 | 8.79E-22 |
| GO0045211 | 24 | postsynaptic membrane | CC | 0.0512891 | 0.011269929 | 1.09E-21 |
| GO0004713 | 55 | protein tyrosine kinase activity | MF | 0.0819382 | 0.019529701 | 3.81E-21 |
| GO0005230 | 24 | extracellular ligand-gated ion channel activity | MF | 0.0514023 | 0.011422827 | 5.63E-21 |
| GO0005249 | 28 | voltage-gated potassium channel activity | MF | 0.054913 | 0.010306306 | 1.80E-20 |
| GO0006464 | 29 | protein modification process | BP | 0.0832241 | 0.015608128 | 9.61E-19 |
| GO0006886 | 125 | intracellular protein transport | BP | 0.092025 | 0.018703284 | 1.37E-18 |
| GO0005525 | 131 | GTP binding | MF | 0.0906426 | 0.017473692 | 1.86E-18 |
| GO0006511 | 51 | ubiquitin-dependent protein catabolic process | BP | 0.0847636 | 0.015903811 | 1.95E-18 |
| GO0003700 | 216 | sequence-specific DNA binding transcription factor activity | MF | 0.0906917 | 0.017729019 | 3.25E-18 |
| GO0005524 | 588 | ATP binding | MF | 0.10988 | 0.023286371 | 4.99E-18 |
| GO0045449 | 88 | regulation of transcription | BP | 0.0894742 | 0.01805865 | 2.48E-16 |
| GO0007264 | 88 | small GTPase mediated signal transduction | BP | 0.0861469 | 0.016854431 | 8.49E-16 |
| GO0055085 | 191 | transmembrane transport | BP | 0.105076 | 0.022459645 | 1.14E-14 |
| GO0008076 | 22 | voltage-gated potassium channel complex | CC | 0.0555557 | 0.009145442 | 1.83E-14 |
| GO0015662 | 22 | ATPase activity, coupled to transmembrane movement of ions, phosphorylative mechanism | MF | 0.0840336 | 0.016416147 | 4.89E-14 |
| GO0007156 | 41 | homophilic cell adhesion | BP | 0.0958267 | 0.022619201 | 6.39E-14 |
| GO0005886 | 39 | plasma membrane | CC | 0.0954627 | 0.022537125 | 9.34E-14 |

Figure S1 **Local GC content distribution of the ground tit (Phumilis), chicken, zebra finch and human genomes.** We used 500-bp non-overlapping sliding windows along the genome.


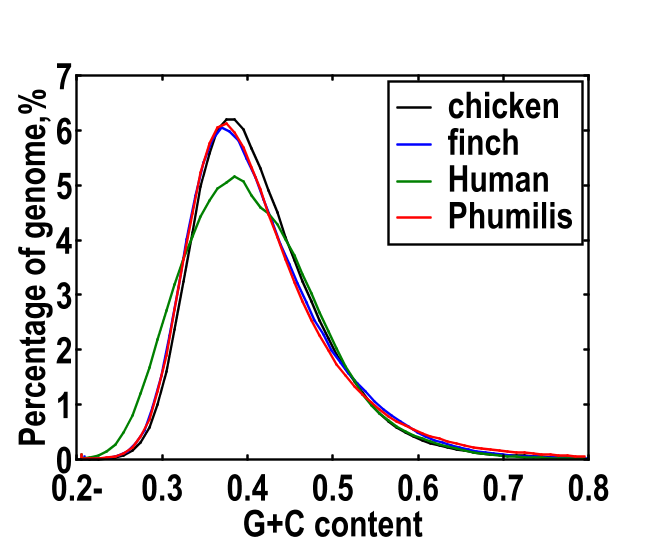


Figure S2 Comparison of gene parameters between ground tit and chicken, zebra finch and human.

Figure S3 **Support evidence of gene models**. “H” Represents gene models supported by homolog information, as well as “P” by de novo prediction and “E” by ESTs.


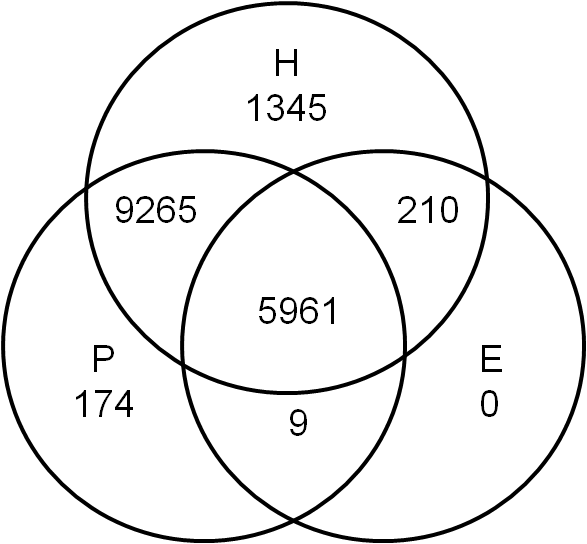


Figure S4 Distribution of ortholog protein identities between chicken and other species for a subset of strictly conserved single-copy orthologs.

Figure S5 **The micro-synteny between genomes of chicken and ground tit**. The X-axis represents the chromosomes of chicken and Y-axis represent the scaffolds of ground tit. The “dot” represents ortholog gene pairs between ground tit and chicken. Two outstanding chromosome inversions in the ground tit were scaled up.


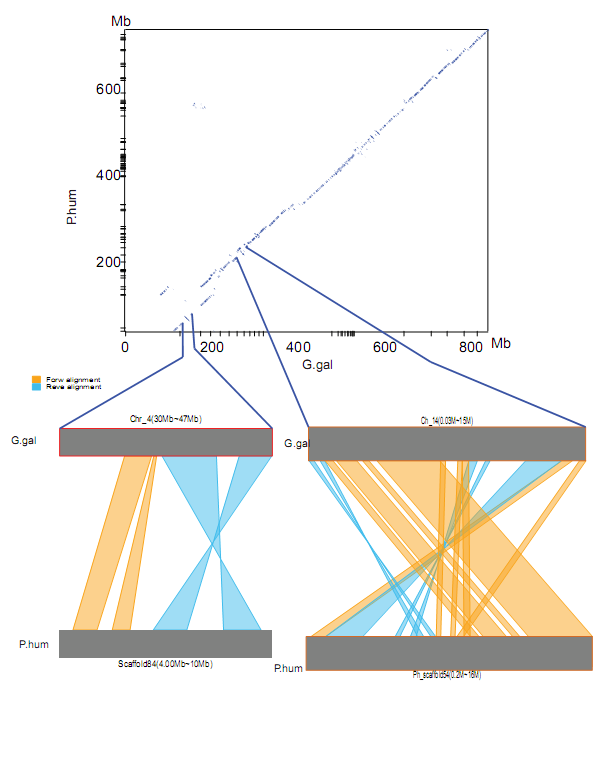


Figure S6 **Venn diagram showing the amount of sequence (in Mbp) aligned among the three avian genomes.** Numbers in brackets refer to the amount of sequence that is not part of the alignments, but as species-specific insertions. For instance, out of the 117 Mbp of the P.humilis genome not aligned to the other two genomes, 19 Mbp are included in the alignments as P.humilis-specific insertions.


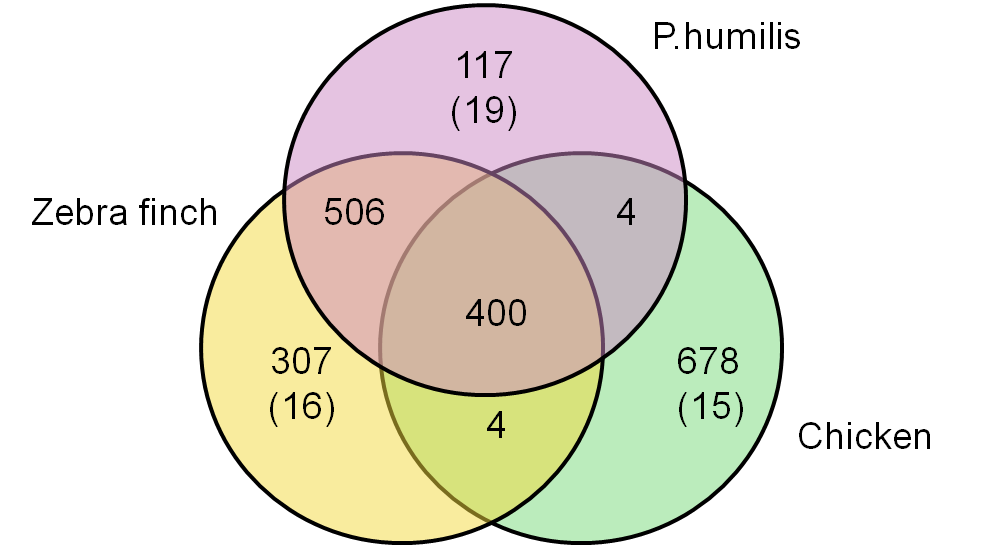


Figure S7 **Dynamic evolution of ortholog gene clusters.** Estimated numbers of orthologue groups in the common ancestral species are shown on the internal nodes. The numbers of orthologous groups that expanded or contracted on each lineage after each speciation is shown on the corresponding branches, with ‘+’ referring to expansion and ‘-’ referring to contraction.
